# AID Activity in B Cells Strongly Correlates with Polyclonal Antibody Affinity Maturation *in-vivo* Following Pandemic 2009-H1N1 Vaccination in Humans

**DOI:** 10.1371/journal.ppat.1002920

**Published:** 2012-09-13

**Authors:** Surender Khurana, Daniela Frasca, Bonnie Blomberg, Hana Golding

**Affiliations:** 1 Division of Viral Products, Center for Biologics Evaluation and Research (CBER), Food and Drug Administration (FDA), Bethesda, Maryland, United States of America; 2 Department of Microbiology and Immunology, University of Miami Miller School of Medicine, Miami, Florida, United States of America; University of Chicago, United States of America

## Abstract

The role of Activation-Induced Cytidine Deaminase (AID) in somatic hypermutation and polyclonal antibody affinity maturation has not been shown for polyclonal responses in humans. We investigated whether AID induction in human B cells following H1N1pdm09 vaccination correlated with *in-vivo* antibody affinity maturation against hemagglutinin domains in plasma of young and elderly individuals. AID was measured by qPCR in B cells from individuals of different ages immunized with the H1N1pdm09 influenza vaccine. Polyclonal antibody affinity in human plasma for the HA1 and HA2 domains of the H1N1pdm09 hemagglutinin was measured by antibody-antigen complex dissociation rates using real time kinetics in Surface Plasmon Resonance. Results show an age-related decrease in AID induction in B cells following H1N1pdm09 vaccination. Levels of AID mRNA before vaccination and fold-increase of AID mRNA expression after H1N1pdm09 vaccination directly correlated with increase in polyclonal antibody affinity to the HA1 globular domain (but not to the conserved HA2 stalk). In the younger population, significant affinity maturation to the HA1 globular domain was observed, which associated with initial levels of AID and fold-increase in AID after vaccination. In some older individuals (>65 yr), higher affinity to the HA1 domain was observed before vaccination and H1N1pdm09 vaccination resulted in minimal change in antibody affinity, which correlated with low AID induction in this age group. These findings demonstrate for the first time a strong correlation between AID induction and *in-vivo* antibody affinity maturation in humans. The ability to generate high affinity antibodies could have significant impact on the elucidation of age-specific antibody responses following vaccination and eventual clinical efficacy and disease outcome.

## Author Summary

Antibody affinity maturation is a key aspect of an effective immune response to vaccines, likely to have an impact on clinical outcome following exposure to pathogens. Activation-Induced Cytidine Deaminase (AID) in B cells is a key enzyme involved in antibody class switching and somatic hypermutation, required for antibody affinity maturation. This human study demonstrated for the first time that induction of AID following H1N1pdm09 influenza vaccination directly correlated with *in-vivo* antibody affinity maturation against the hemagglutinin globular domain (HA1), containing most of the protective targets. Importantly, age differences were found. In younger adults, significant affinity maturation to the HA1 globular domain was observed, which associated with higher initial levels of AID and >2-fold-increase in AID after vaccination. With increased age, a drop in AID activity post-vaccination correlated with lower affinity maturation of the polyclonal antibody responses against the pandemic influenza HA1. However, in a subset of elderly (>65 yr), high affinity antibodies against the HA1 were present prior to vaccination but, in the absence of AID, did not undergo further maturation. Therefore, vaccination of divergent human populations, especially older individuals, should take into consideration their individual AID status and the history of exposure and vaccination against the specific pathogen.

## Introduction

Antibody affinity maturation is a key aspect of an effective immune response to vaccines likely to provide a significant protection against human pathogens. The discovery of Activation-Induced Cytidine Deaminase (AID) has led to the elucidation of key molecular mechanisms involved in class switch recombination (CSR) and somatic hypermutation (SHM), which occur in B cells as they mature in germinal centers of lymph nodes and spleen in response to antigenic stimulation and T cell signals [1]–[2]. Recently, using mouse models of AID-dependent cell labeling, it was shown that memory B cells appear in the IgM^+^ and IgG^+^ subsets both in germinal centers and outside of B cell follicles. After challenge, the IgG^+^ memory B cells differentiate into plasma cells, whereas the IgM^+^ memory B cells reinitiate a germinal center reaction, resulting in class switching and SHM leading to production of higher affinity BCR expressed on memory and plasma cells [3].

Age-related defects in B cells have been reported and these include decrease in AID expression due to impairment of the transcription factor E47 which activates AID [4]. Recirculating B cells found in the peripheral blood can be used to measure response under *in vitro* conditions that may partially reflect the *in vivo* vaccine-induced immune response of the individual. Following polyclonal or antigen-specific stimulation *in vitro*, AID expression was shown to increase in B cells from young individuals, but significantly less in B cells from elderly individuals. This up-regulation of AID expression occurs primarily in stimulated naïve and IgM^+^ memory, but not in switched memory B cells [4], [5], [6]. More recently, in two studies on age effects on influenza vaccination, we have shown that levels of AID in vaccine-stimulated B cells and serum antibody responses are positively correlated in humans [6]–[7]. This correlation suggested that a defect in AID induction in PBMC derived B cells (measured *in vitro*) correlated with the observed decrease in the number of switched memory B cells and a reduced number of IgG plasmablasts after vaccination *in vivo* [6]–[7]. While association between CSR and AID has been suggested before, no information to date is available for the direct involvement of AID and SHM and/or antibody affinity maturation in humans.

Influenza subtypes are classified based on the antigenic variation within influenza hemagglutinin (HA) as measured by a hemagglutination inhibition (HI) assay. The HI assay is dependent on antibodies that inhibit the interaction between the sialic acid receptor on red blood cells (RBC) and the receptor binding domain (RBD) within the globular domain (HA1) of influenza hemagglutinin. Therefore, the antigenic differences within influenza viruses are primarily due to mutations within the HA1 domain, while the protein sequence within the HA2 stalk domain is highly conserved among multiple influenza subtypes and strains. Human polyclonal responses against one subtype can show significant cross-reactivity to the hemagglutinin of other subtypes due to this high sequence conservation in the HA2 domain as previously shown [8], [9], [10]. But this binding cross-reactivity does not commonly translate into cross protection, since most of the antibodies against the HA2 stalk do not block virus infectivity. In our previous studies, we have demonstrated that most of the polyclonal neutralizing antibody responses following influenza infections or inactivated subunit vaccination, as measured in HI or microneutralization (MN) assays, targeted the HA1 domain [8], [11]. Recently, rare antibodies with broad neutralizing cross-reactivity that target the HA2 stem were reported, but they are not easily elicited by traditional vaccination [12]. Therefore it is important to study the humoral responses against the different domains within the influenza hemagglutinin that were shown to evolve independently for the HA2 and HA1 antigenic regions [10].

The involvement of AID in CSR has been shown. However, the involvement of AID in somatic hyper mutation (SHM), leading to antibody affinity maturation, is poorly understood especially for polyclonal antibody responses in humans. In the current study, we correlated for the first time the levels of AID in peripheral B cells and AID fold increase post-H1N1pdm09 vaccination with the *in vivo* humoral immune responses against the H1N1pdm09 inactivated influenza vaccine in individuals ranging in age from 20 to 90 years. We measured polyclonal antibody affinity of human plasma to the globular domain (HA1) and the conserved stalk domain (HA2) of the H1N1pdm09 hemagglutinin using a Surface Plasmon Resonance (SPR) based real-time kinetics assay as previously described [9], [10]. Changes in polyclonal antibody-antigen complex dissociation rates, as indicators of antibody affinity maturation in human plasma following vaccination were correlated with fold-increases in AID levels of B cells after vaccination and with AID levels in B cells before vaccination. These results demonstrate for the first time that AID contributes to polyclonal antibody affinity maturation in response to influenza vaccination in humans.

## Results

### Age-dependent decrease in AID mRNA following H1N1pdm09 vaccination

Forty two individuals, 20–90 year old, were enrolled in the H1N1pdm09 vaccine trial. HI titers and AID mRNA expression were evaluated at day 0 (t0) and day 28 (t28) (before vaccination and 28 days after vaccination). All the individual data are presented in Table S1.

Only 3/26 young and 2/16 elderly individuals had non-protective titers at t0 (below 1∶40). Moreover, 2/26 young and 3/16 elderly had higher HI titers at t0 (1∶160–1∶640), reflecting possible recent exposure to the first wave of the pandemic H1N1pdm09. Importantly, vaccination induced a significant boost in HI titers that was more robust in young compared with elderly individuals (average fold-increase of 13 in young versus 4 in elderly individuals, respectively, p = 0.004) (Table S1). Although it has been reported that archived plasma samples from elderly ≥80 years of age have increased neutralization titers to the H1N1pdm09 [13], the initial titers in young and elderly individuals in our cohorts were not significantly different (reciprocal titers at t0: 74±12 and 108±39 in young versus elderly, respectively, p = 0.44). Therefore, the response parameters measured, including AID fold-increase at t28, are not likely to be impacted by difference in initial (t0) HI titers, which reflect long lived plasma cells.

We have previously shown that the intrinsic defect in AID expression in B cells from the elderly population is a general phenomenon, and therefore not limited to influenza specific cells [14] [4]. In the current study, we have used the *in vitro* AID response as a biomarker for an effective influenza-specific B cell response. The inactivated vaccine used for vaccination of the subjects was also used for *in vitro* stimulation at t0 and t28. This approach is supported by our earlier studies demonstrating a strong correlation between AID inducibility in peripheral B cells *in vitro* with the *in vivo* serum HI responses [6]–[7]. A two fold change (or higher) in AID mRNA level was empirically determined as a positive response to the vaccine and was found to correlate with positive HI responses [4]–[6].

**Figure 1:**
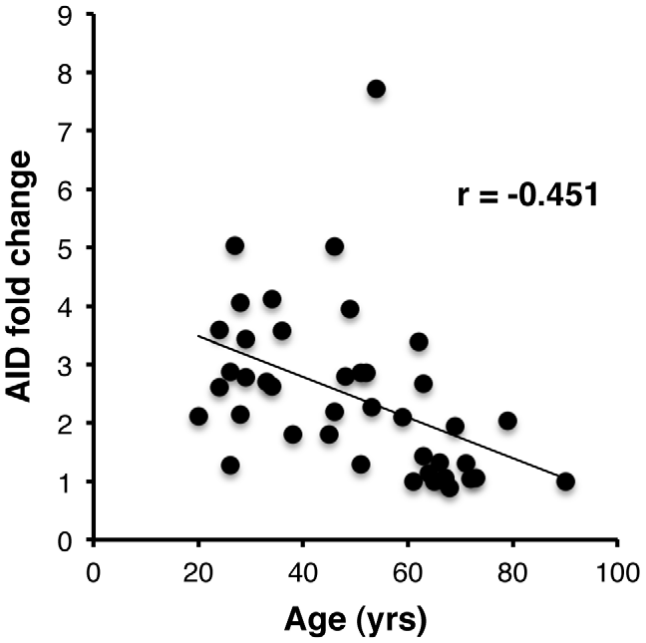
AID response following H1N1pdm09 vaccination declines in age-dependent manner. PBMC (10^6^ cells/ml), were isolated from 42 individuals before (t0) and after H1N1pdm09 vaccination (t28), and were cultured with vaccine for 7 days. At the end of culture, cells were processed as described in Materials and Methods. Results are expressed as qPCR value fold-increase in AID mRNA expression (normalized to GAPDH mRNA) after vaccination compared with the age of the vaccinated individuals. The fold-increase in AID mRNA expression after vaccination is calculated as follows: qPCR values after H1N1pdm09 vaccination/qPCR values before vaccination. AID fold change ≥2 is considered meaningful. A statistically significant negative correlation coefficient was observed between levels of AID induction with increase in age. Representative of two experiments.

Results shown in Table S1 and Fig. 1 demonstrate lower fold-increase in AID mRNA levels in peripheral B cells following H1N1pdm09 vaccination in elderly individuals (3/16; 19%, with AID fold change ≥2) compared with the younger adults with 22/26 individuals (85% showing ≥2 fold increase in AID mRNA expression at t28). A statistically significant negative correlation coefficient was observed between levels of AID induction and age (Fig. 1). Furthermore, a positive correlation was observed between fold increase in AID induction (t28/t0) and the fold increase in HI titers (Table S1), confirming our previous results showing that the *in vitro* AID mRNA increase in vaccine-stimulated B cells positively correlated with the influenza vaccine-specific HI titers [6], [7].

These results are not due to higher numbers (and their phenotypes) or the percentages of memory B cells (both switched memory and IgM memory) in elderly individuals, as we have previously demonstrated that these do not increase with age. Since AID induction occurs primarily in naïve and IgM memory cells, the findings in the current cohort confirm our previous observations on the age-related decline in AID induction in peripheral B cells, and are not simply a reflection of a decrease in the number of responding B cells. Also, this cannot be due to a difference in kinetics in B cell responses, because we have done kinetics previously in young and elderly and found the peak response to be coincident and optimal at t28 for both age groups [6].

### Age-related polyclonal antibody affinity maturation in human plasma to the HA1 globular domain (but not to the HA2 stalk domain) following H1N1pdm09 vaccination

**Figure 2:**
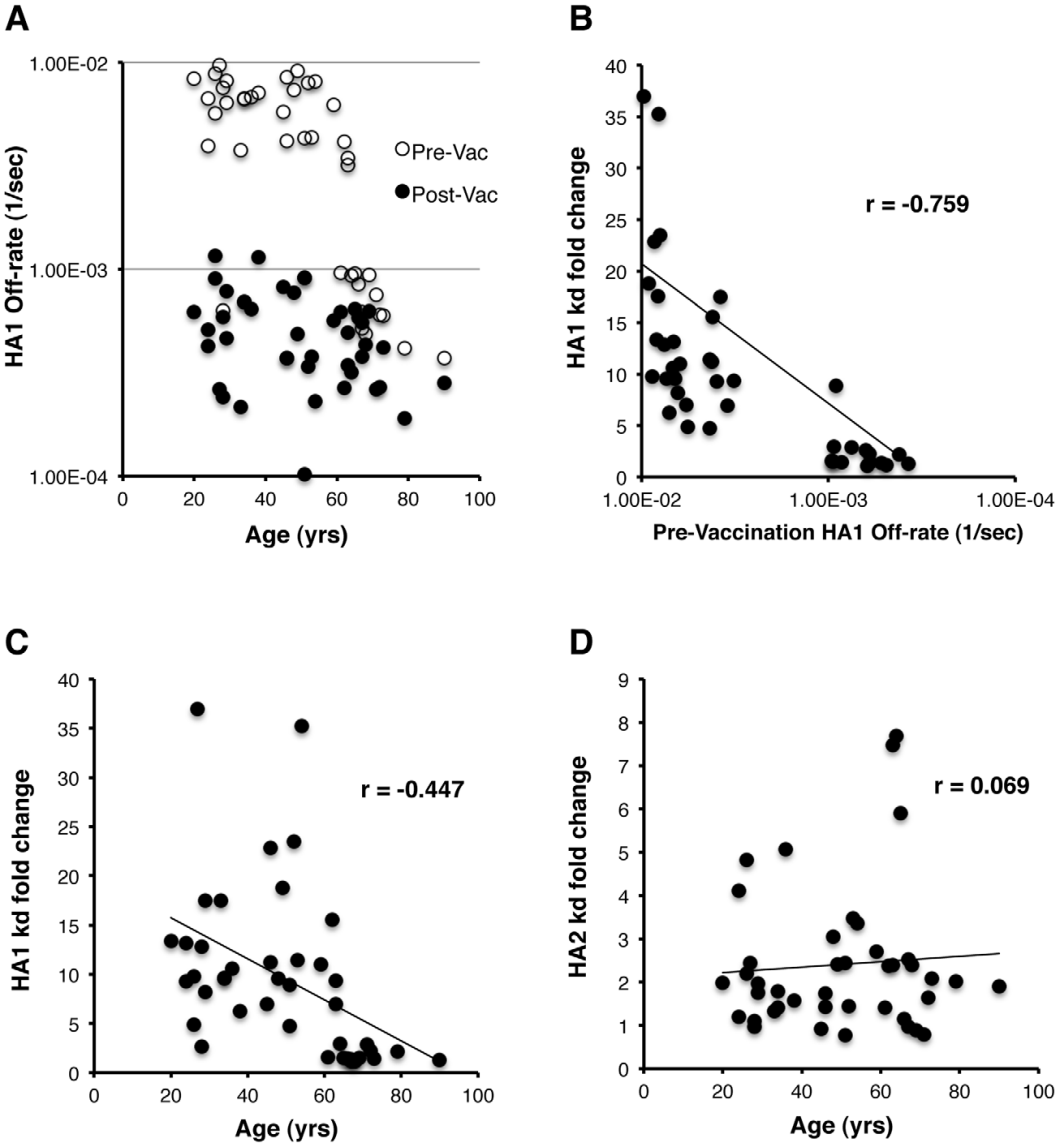
Antibody affinity maturation, measured by the antigen-antibody complex dissociation rate, increases for HA1 domain (but not HA2) following immunization with H1N1pdm09 subunit vaccine. (A) SPR analysis of pre- (open circles) and post- (filled circles) H1N1pdm09-vaccinated human plasma from all study participants (20–90 yrs old). Binding assays were conducted with functional H1N1pdm09 HA1 (1–330) or HA2 stalk domains [34]. Polyclonal human plasma antibody off-rate constants were determined as previously described [9]. (B) Correlation between increase in polyclonal plasma antibody affinity to HA1 domain (measured by a fold decrease of the antigen-antibody complex dissociation-rate) following H1N1pdm09 vaccination compared with the pre-vaccination off-rates against HA1. (C, D) Fold-increase in human plasma antibody affinity (decrease in antibody off-rate) after vaccination against the globular HA1 domain (C) or HA2 stalk domain (D) compared with the age of the vaccinated individuals. Post-H1N1pdm09 vaccination increase in polyclonal antibody affinity to the HA1 domain (but not HA2) was inversely correlated with subjects' age.

To determine the effect of age on antibody affinity maturation in human plasma against H1N1pdm09-HA following vaccination, polyclonal antibody off-rate constants, which describe the strength of antigen-antibody complexes, were determined directly from the antibody interactions with the HA1 globular domain or HA2 stalk domain, using SPR as described before [9]. We have previously established that the antigen-antibody dissociation kinetics is not influenced by the antibody concentration in the polyclonal sera but reflects the avidity of bound antibodies for the proteins on the chip surface. For each individual, the dissociation off-rates (Kd) were measured for pre-vaccination day 0 (t0) and post-vaccination day 28 (t28) samples (Fig. 2A), and fold-change in Kd to the HA1 (Fig. 2B–C) and HA2 domains (Fig. 2D) were calculated. Positive fold change of antibody off-rates (post/pre-vaccination values) indicates slower dissociation of antigen-antibody complexes reflecting increased polyclonal antibody affinity in the plasma of vaccinated individuals. Since the HA1 globular domain is not as conserved as HA2, the response to HA1 better estimates the newly generated strain-specific antibodies, and is better suited for measurements of affinity maturation following H1N1pdm09 vaccination. In most subjects, following vaccination, the anti-HA1 antibody dissociation rates decreased (indicating increased antibody affinity) by ≥1 log for most vaccinated individuals, reaching off-rates of 10^−3^–10^−4^ per sec (Fig. 2A; filled vs. open circles). Interestingly, in a significant fraction of older adults (≥65 years), the pre-vaccination (t0) anti-HA1 antibody binding off-rates were significantly slower (<10^−3^/sec) in the plasma compared with the rest of the cohort (>10^−3^/sec), demonstrating the presence of high affinity antibodies against the H1N1pdm09 HA1 prior to vaccination (Fig. 2A, open circles) in that age group.

Our data also demonstrated an inverse correlation between the fold-change in anti-HA1 antibody affinity following H1N1pdm09 vaccination and the pre-vaccination antibody off-rates to the HA1 globular domain (Fig. 2B). Importantly, the fold increase in polyclonal antibody affinity following H1N1pdm09 vaccination (t28/t0) to the HA1 domain, as measured by slower off-rates, declined with age (Fig. 2C).

In contrast to the findings with HA1 coated chips, the affinity of polyclonal antibody binding to the HA2 stalk domain, which is highly conserved between the H1N1pdm09 and seasonal H1N1 influenza strains, was uniformly high (off-rate constant ∼10^−4^/sec) even before vaccination, and the fold change in anti-HA2 antibody off-rates (t28/t0) of human plasma did not demonstrate any age dependence (Fig. 2D).

### Anti-HA1 polyclonal antibody affinity maturation in human plasma strongly correlates with the increase in AID expression in human B cells following H1N1pdm09 vaccination

**Figure 3:**
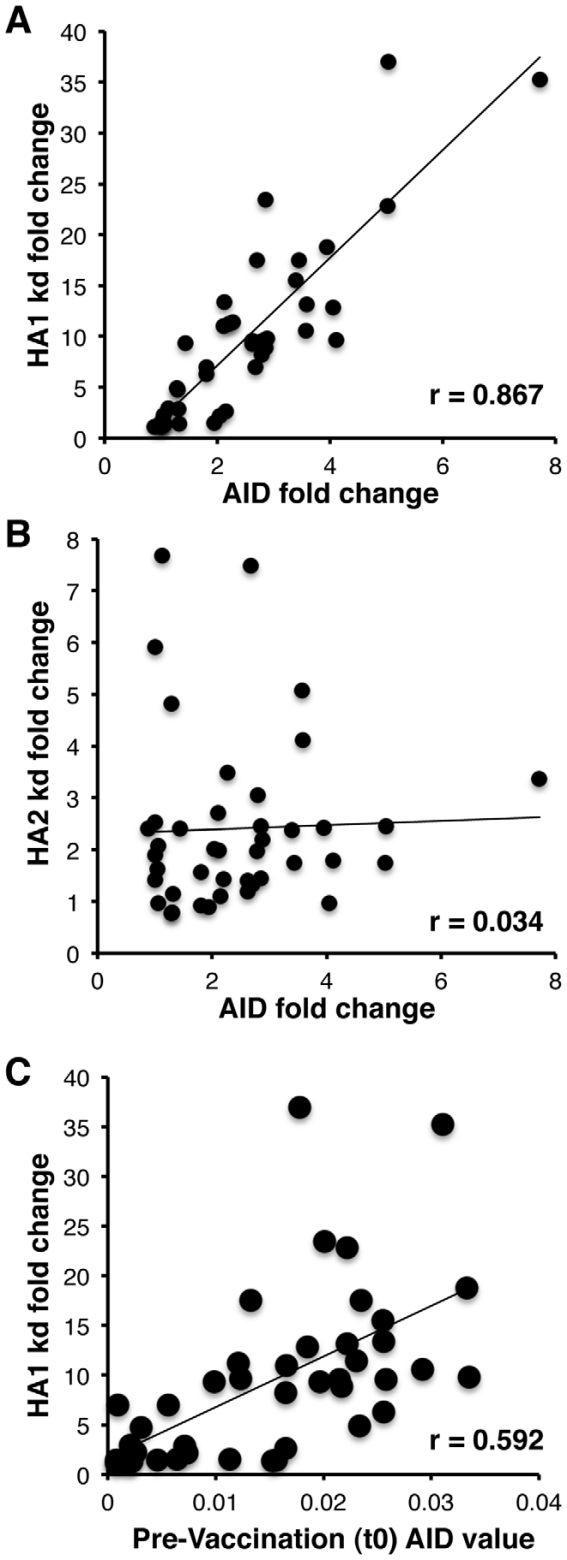
Polyclonal human plasma antibody dissociation rates for HA1 correlate with AID induction in influenza specific B cells. Human polyclonal antibody off-rate constants that describe the fraction of antibody-antigen complexes decaying per second were determined directly from the plasma sample interaction with H1N1pdm09-HA1 (A) and H1N1pdm09-HA2 (B) proteins during the dissociation phase for pre- and post-vaccination samples from each individual. Fold change in plasma antibody off-rate constants (each symbol represents one individual) were determined as described in Materials and Methods. (A) The fold decrease in antibody off-rates following H1N1pdm09 vaccination strongly correlated with AID fold-change. (B) No significant correlation was observed in antibody affinity against HA2 and AID fold change. (C) Positive correlation between AID activity in B cells before vaccination (t0) and fold change in antibody affinity in t28 vs. t0. The off-rate constants were determined from two independent SPR runs.

To determine if AID induction following H1N1pdm09 vaccination correlates with the change in antibody affinity against HA1 and HA2 domains of H1N1pdm09-HA, the increase in AID expression in stimulated B cells following H1N1pdm09 vaccination (t28/t0) was correlated with the fold change in antigen-antibody complex off-rate binding constants to the HA1 globular domain (Fig. 3A) or HA2 stalk domain (Fig. 3B), for each vaccinated individual. As can be seen in Fig. 3, a strong direct correlation was demonstrated between the fold increase in AID and the increase (fold change) of antibody affinity to the HA1 domain (r = 0.867) (Fig. 3A). A significant positive correlation was also observed between AID levels at t0 and fold increase in antibody affinity to the HA1 domain (Fig. 3C), indicating that both AID levels in B cells at t0 and the capacity of B cells to up-regulate AID expression during an antigen-specific response are likely contributors to antibody affinity maturation *in-vivo*. In contrast, no correlation between the change in antibody binding affinity to the conserved HA2 stalk domain and AID activity was observed (Fig. 3B).

## Discussion

The involvement of AID in class switch recombination (CSR) has been shown [15], [16]. However, the role of AID in somatic hypermutation and antibody affinity maturation has only been postulated in humans, and may be extremely important in understanding age-related antibody responses following vaccination and their impact on vaccine efficacy and disease outcome.

Germinal centers (GC) in lymph nodes and spleen were shown to be the key anatomical sites where B cells undergo class-switch and affinity maturation in AID-dependent mechanisms after exposure to foreign antigen. The GC formation depends on close interactions between antigen specific B and follicular helper T cells (T_FH_) [17]–[18] [19], [20]. In humans it is very difficult to access the lymph nodes for large scale studies. However, there is evidence that circulating peripheral B cells could be used to measure AID mRNA baseline levels and induction after vaccination, thus mimicking the earlier GC reactions. We have previously shown that AID can be induced in cultures of B cells stimulated not only with anti-CD40/CD40L plus cytokines [14] [4], but also with vaccines and CpG [6]–[7]. Thus, the response of peripheral B cells to vaccine stimulation *in vitro* in the presence of co-stimulatory signals provided by T cell signals or TLR agonist seems to mimic the early germinal center reactions. Using AID mRNA induction as a parameter of vaccine responsiveness, we have previously shown that the *in vivo* and *in vitro* response of B cells to the influenza vaccine are both decreased with advanced age with strong correlation, providing support for the use of AID induction in peripheral B cells as a biomarker of *in vivo* immune responsiveness [6]–[7]. In previous studies we have compared the response of purified B cells with PBMC and the AID fold-increase to the vaccine is comparable. Therefore, the contribution of T cells to this AID response is not significant [7].

In the current study we demonstrate for the first time a strong correlation between baseline AID levels as well as fold-induction of AID activity following vaccination and polyclonal antibody affinity maturation measured by SPR in plasma following influenza vaccination in humans. We also demonstrate the presence of pre-existing high affinity antibodies to the pandemic H1N1 HA1 globular domain in a fraction of older adults (65–90 yr) in agreement with our findings in another H1N1pdm09 vaccine trial [10].

On the other hand, the affinity of polyclonal antibody binding to the H1N1pdm09 HA2 stalk domain was high even before vaccination (∼10^−4^/sec) in all individuals, irrespective of age, and did not increase post-vaccination. The high conservation of HA2 protein sequence between H1N1pdm09 and circulating seasonal H1N1 influenza strains most likely have resulted in maximal affinity maturation of HA2-binding antibodies. Therefore, even in younger individuals with significant induction of AID expression post vaccination, there was no significant increase in the affinity of anti-HA2 antibodies in human plasma. These findings suggest that there is an upper limit of antibody affinity for human polyclonal antibodies *in-vivo*.

In a recent study on B cell responses to seasonal Trivalent Inactivated Vaccine (TIV), it was found that the frequency of vaccine-specific plasmablasts were lower in the elderly than in young adults. However, no clear difference in vaccine-specific affinity of plasmablast derived polyclonal or monoclonal antibodies against complete hemagglutinin (HA0) was found between younger and older individuals [21]–[22]. These findings are of particular importance, since in these studies the entire HA vaccine was used to measure total antibody secreting cells (ASC) and binding avidity of antibodies. Due to the high conservation of HA2, the level of cross-reactive binding to the stalk domain could have masked significant differences in the avidity of binding to the HA1 domain among different age groups. Moreover, AID activity was not measured in the previous study and the role of AID in promoting antibody affinity maturation in polyclonal B cell responses in humans following vaccination was not understood.

In other studies, the frequency of mutations in Ig V_H_/V_L_ genes in peripheral and tonsillar B cells of older people was increased when compared to younger individuals[23]–[24], but this did not correlate with AID expression levels, suggesting that the hypermutated Ig V_H_/V_L_ genes were from long lived (affinity matured) memory B cells and plasma cells.

The affinity of antibodies is likely to play a key role *in vivo*, especially very early in infection (i.e., unfavorable antibody/viral load ratios). During the H1N1pdm09 pandemic, low affinity antibodies in some infected individuals were associated with more severe disease [25]. The high affinity anti-HA1 antibodies found in some older individuals (≥65 years) prior to vaccination most likely represent long term plasma cells or memory IgG^+^ B cells that have undergone affinity maturation through exposure to H1N1pdm09 like influenza virus strains that circulated in the US during the first half of the 20^th^ century.

Importantly, the significant differences in binding affinity found between the older vs. younger adults was only demonstrated for antibodies targeting the HA1 globular domain but not the HA2 stalk domain. The presence of higher affinity antibodies to HA1 in older individuals pre-vaccination provides an additional explanation for the unusually low rate of severe respiratory disease during the 2009 H1N1 pandemic in this age group that is usually most susceptible to morbidity and mortality due to seasonal influenza.

Thus, the present study demonstrated that an increase in AID expression was associated with affinity maturation of polyclonal antibodies in human plasma. In the older individuals, AID induction was very low. However, in spite of low AID activity and minimal change in and HI titers, post-H1N1pdm09 vaccination, older adults were relatively protected from the pandemic H1N1 compared with younger adults probably due to the presence of affinity mature B cells and polyclonal antibody responses [26], [27], [28], [29], [30].

We hypothesize that the antibody responses to the influenza vaccine during the 2009 and 2010 seasons in elderly (in terms of antibody affinity) will not be the norm when novel influenza strains are circulating, which is the more common scenario. The ability to generate new high affinity antibodies from naïve B cells will require AID activation following exposure to neoantigens such as drifted seasonal influenza strains, avian influenza (H5N1, H7N7), or emerging pathogens, putting the older individuals at a disadvantage [31], [32]. Therefore, vaccination of divergent human populations, especially older individuals, should take into consideration the AID status and the history of exposure and vaccination against the specific pathogen. Targeted vaccination of older adults (e.g., against varicella reactivation and other pathogens) is likely to be more effective before the precipitous drop in AID activity.

## Methods

### Subjects

Experiments were conducted using blood isolated from healthy volunteers of different ages after appropriate signed informed consent. The study has been approved with IRB protocol #20070481. Participants included 26 young subjects (age 20–59 years) and 16 elderly (age 60–90 years). Of these, 22 young and 12 elderly were vaccinated using the H1N1pdm09 monovalent vaccine (from Novartis) in the 2009–10 season, whereas 4 young and 4 elderly were vaccinated with the trivalent inactivated vaccine (TIV from GSK), containing the H1N1pdm09 strain during the 2010–2011 season. Blood samples were collected before (t0) and 4 weeks (t28) post-vaccination. Subjects have not received the H1N1 vaccine or had flu-like symptoms at the time of enrollment.

### Hemagglutination inhibition (HI) assay

Immune responses to the H1N1pdm09 vaccine in young and elderly individuals at t0 and t28 were measured by HI assay, as previously described [6] [33].

### PBMC cultures

PBMC were collected by density gradient centrifugation on Ficoll–Paque premium solution (GE Healthcare). Cells were then washed three times with PBS and frozen. Frozen PBMC were thawed in a 37°C water bath and washed twice with medium (RPMI 1640), resuspended, rested for 1 h, and then counted in trypan blue to evaluate cell viability. PBMC (10^6^/ml; ≥80% viability) were cultured in complete medium (RPMI 1640, supplemented with 10% FCS, 10 µg/ml Pen-Strep, 1 mM Sodium Pyruvate, 2×10^−5^ M 2-ME and 2 mM L-glutamine). PBMCs from individuals in the 2009–10 season were stimulated with H1N1pdm09 monovalent vaccine (2 µl/10^6^ cells). PBMC from the individuals in the 2010–11 season were stimulated with the H1N1pdm09 egg-derived mono-bulk subunit antigens (A/California/07/2009; Novartis Vaccines and Diagnostics, Siena, Italy) at the same concentration. Although B cells in the PBMC cultures have been stimulated in the presence of other cell types (primarily T cells and monocytes), our endpoint is to measure a B-cell response and AID is expressed exclusively in B cells. In previous studies, we have compared the response of purified B cells and PBMC cultures and demonstrated that the AID mRNA fold-increase in response to vaccine stimulation in both *in vitro* cell cultures was comparable [7].

At the end of stimulation, cells were harvested and mRNA extracted using the μMACS mRNA isolation kit (Miltenyi Biotech) according to the manufacturer's protocol, eluted into 75 µl of elution buffer, and stored at −80°C until use. qPCR was performed as previously described [5]. We always assay the same number of B cells in PBMC cultures of young and elderly individuals. This was confirmed by measuring B cell subsets (numbers and frequencies) using flow cytometry. Furthermore, we measure the fold-increase from day 0 (t0) to day 28 (t28) of the AID response generated in cultures, since the numbers of B cells are maintained between these two time points as determined by flow cytometry. Moreover, we have shown that the subsets which make AID *in vitro* are the naïve and IgM memory B cells [4], [14], which are present in similar percentages in young and elderly individuals [5].

### Quantitative PCR (qPCR)

Reactions were performed with Taqman Master mix and primers, as described [4], [6]. The number of cycles at which transcripts reached a cycle threshold (Ct) for AID and GAPDH as control was determined and used to calculate ΔCt (target gene cycles relative to GAPDH cycles). A two-fold rise in AID mRNA, indicates a positive response to the vaccine with the t28/t0 values reporting the fold rise in AID mRNA [4] [5], [6]. We have conducted a series of validation experiments including titration (5-fold serial dilutions) of cDNA used as template in the qPCR assay, and found that the ratio of AID/GAPDH does not change irrespective of the cDNA template used in the qPCR. We validate the qPCR conditions at the beginning of each flu season using the relevant vaccine strains. We also report the AID mRNA values at t0, which reflect the functional baseline level for each subject at the time of vaccination.

### Affinity measurements by Surface Plasmon Resonance

Steady-state equilibrium binding of pre- and post-H1N1 human vaccine plasma polyclonal antibodies was monitored at 25°C using a ProteOn SPR biosensor (BioRad). In the SPR assays described in this study, we are only measuring the antibody off-rate constants which describe the stability of the complex. The fraction of antigen-antibody complexes decaying per second were determined directly from plasma sample interaction with properly folded, H1N1pdm09 functional HA1 globular domain and HA2 stalk domain proteins [34] during the dissociation phase. We have previously demonstrated that the antigen-antibody dissociation kinetics were independent of the antibody concentration in the human polyclonal sera/plasma. In these SPR assays serial dilutions of the polyclonal sera (10, 33, 100) were run on the HA coated protein chip surface. The kinetics of dissociation were identical as indicated by parallel lines in the dissociation phase and hence independent of serum antibody concentration [9], [10]. Antibody off-rate constants were calculated using the BioRad ProteOn manager software for the heterogeneous sample model as previously described [9]. To improve measurements, the off-rate constants were determined from two independent SPR runs.

## Supporting Information

Table S1 **Distribution of age, pre and post-vaccination HI antibody titers and AID mRNA in H1N1pdm09 vaccinated subjects.**
(PDF)
