# Supplementary material for: AID Activity in B Cells Strongly Correlates with Polyclonal Antibody Affinity Maturation in-vivo Following Pandemic 2009-H1N1 Vaccination in Humans
Source: PLoS Pathog. 2012 Sep 13;8(9):e1002920. doi: 10.1371/journal.ppat.1002920 (PMC3441753; doi:10.1371/journal.ppat.1002920)
Supplement: Table S1 — Distribution of age, pre and post-vaccination HI antibody titers and AID mRNA in H1N1pdm09 vaccinated subjects. (PDF) [file ppat.1002920.s001.pdf]

Table S1- Distribution of age, pre and post-vaccination HI antibody titers and AID mRNA in H1N1pdm09 vaccinated subjects

Subject	Age	H1N1pdm09-specific HI			H1N1pdm09-specific AID		
	(Yrs)	Day 0 (t0)	Day 28 (t28)	Fold-inc	Day 0 (t0)	Day 28 (t28)	Fold-inc
H0920	20	80	1280	16	0.0256	0.0541	2.11
H0908	24	80	640	8	0.0222	0.0797	3.59
H0914	24	160	2560	16	0.0197	0.0515	2.62
H0904	26	20	80	4	0.0234	0.0300	1.28
H0905	26	80	2560	32	0.0335	0.0963	2.88
H0913	27	320	1280	4	0.0178	0.0896	5.03
H0909	28	80	1280	16	0.0185	0.0750	4.05
F1011	28	10	40	4	0.0165	0.0354	2.15
H0902	29	80	320	4	0.0133	0.0369	2.78
H0917	29	80	1280	16	0.0165	0.0568	3.44
F1012	33	80	320	4	0.0235	0.0635	2.70
H0935	34	40	320	8	0.0258	0.1061	4.11
H0943	34	40	160	4	0.0123	0.0323	2.62
H0934	36	80	1280	16	0.0292	0.1043	3.57
H0903	38	80	1280	16	0.0256	0.0462	1.80
H0941	45	40	80	2	0.0056	0.0101	1.80
H0932	46	40	640	16	0.0121	0.0607	5.02
H0942	46	40	160	4	0.0222	0.0488	2.20
H0940	48	80	1280	16	0.0215	0.0600	2.79
H0936	49	40	640	16	0.0333	0.1315	3.95
H0937	51	40	80	2	0.0031	0.0040	1.29
F1037	51	20	320	16	0.0217	0.0619	2.85
H0918	52	80	1280	16	0.0201	0.0574	2.85
H0929	53	80	5120	64	0.0231	0.0525	2.27
F1003	54	80	320	4	0.0311	0.2400	7.72
H0912	59	80	320	4	0.0166	0.0350	2.11
H0931	61	40	80	2	0.0064	0.0064	1.00
H0915	62	160	1280	8	0.0255	0.0866	3.39
H0907	63	320	640	2	0.0009	0.0013	1.44
H0919	63	80	1280	16	0.0099	0.0264	2.67
H0921	64	40	80	2	0.0021	0.0024	1.13
H0930	65	40	40	1	0.0008	0.0008	1.01
H0901	66	80	160	2	0.0046	0.0061	1.32
F1007	67	40	160	4	0.0018	0.0018	1.00
F1038	67	20	160	8	0.0153	0.0162	1.06
H0933	68	640	640	1	0.0008	0.0008	1.00
H0911	69	40	160	4	0.0113	0.0219	1.94
H0923	71	40	80	2	0.0071	0.0093	1.31
H0916	72	40	40	1	0.0026	0.0027	1.05
H0925	73	40	320	8	0.0156	0.0165	1.06
F1009	79	80	320	4	0.0073	0.0149	2.03
F1010	90	20	80	4	0.0022	0.0022	1.00
